# Difference in Motor Fatigue between Patients with Stroke and Patients with Multiple Sclerosis: A Pilot Study

**DOI:** 10.3389/fneur.2014.00279

**Published:** 2014-12-22

**Authors:** Aida Sehle, Manfred Vieten, Annegret Mündermann, Christian Dettmers

**Affiliations:** ^1^Division of Sport Science, University of Konstanz, Konstanz, Germany; ^2^Lurija Institute, Kliniken Schmieder Allensbach, Allensbach, Germany; ^3^Department of Orthopaedics, University Hospital Basel, Basel, Switzerland; ^4^Kliniken Schmieder Konstanz, Konstanz, Germany

**Keywords:** multiple sclerosis, stroke, motor fatigue, gait analysis, attractor, fatigue index, questionnaire assessment, physical performance

## Abstract

Fatigue is often reported in stroke patients. However, it is still unclear if fatigue in stroke patients is more prominent, more frequent or more “typical” than in patients with multiple sclerosis (MS) and if the pathophysiology differs between these two populations. The purpose of this study was to compare motor fatigue and fatigue-induced changes in kinematic gait parameters between stroke patients, MS patients, and healthy persons. Gait parameters at the beginning and end of a treadmill walking test were assessed in 10 stroke patients, 40 MS patients, and 20 healthy subjects. The recently developed Fatigue index Kliniken Schmieder (FKS) based on change of the movement’s attractor and its variability was used to measure motor fatigue. Six stroke patients had a pathological FKS. The FKS (indicating the level of motor fatigue) in stroke patients was similar compared to MS patients. Stroke patients had smaller step length, step height and greater step width, circumduction with the right and left leg, and greater sway compared to the other groups at the beginning and at the end of test. A severe walking impairment in stroke patients does not necessarily cause a pathological FKS indicating motor fatigue. Moreover, the FKS can be used as a measure of motor fatigue in stroke and MS and may also be applicable to other diseases.

## Introduction

Fatigue is a frequent symptom in many neurologic diseases (1) and especially common and disabling in patients with multiple sclerosis (MS) (2, 3). Moreover, fatigue is often the reason for early retirement and hence represents a high economic burden (4). Despite the high prevalence of fatigue in MS of up to 83% (1), its pathophysiology is largely unknown (5, 6). Nonetheless, several pathophysiological pathways have been proposed: demyelinisation and axonal injury may cause “electric failure” (7); immunological and inflammatory factors such as cytokines may hamper neuronal processing (8); hormonal dysregulation may be caused by failed cortico-hypothalamic loops (9); and reorganization and compensation might add to the ineffectiveness of cerebral control and cause fatigue (10). Moreover, fatigue may be secondary to conditions including depression, sleep disorders, physical deconditioning, anemia, or side effects of medication (3, 11, 12).

In the last decade, reports of fatigue in neurological conditions other than MS, such as for instance stroke, have become more frequent (13, 14), and the prevalence of fatigue in patients after stroke ranges from 36 to 77% (1). Fatigue is a common and debilitating symptom even in patients with good recovery after stroke (15). Patients’ level of fatigue does not change over time (16) and baseline fatigue immediately after a stroke predicts fatigue outcome (17). Staub and Bogousslavsky (18) suspected that primary poststroke fatigue may be caused by minor attentional deficits due to the interruption of neural networks, such as the reticular activating system. Patients use different strategies and coping styles to deal with poststroke fatigue (19). In addition, poststroke fatigue appears to be an independent determinant of not being able to resume paid work following stroke (20).

Currently, there are no widely accepted standard definitions or accepted standardized methods and instruments for assessing fatigue (1). Moreover, fatigue is understood as a multidimensional phenomenon with different aspects including a complex interplay between the underlying disease process, peripheral, and central control systems, as well as environmental factors (21). Its multidimensionality complicates the assessment of fatigue in neurological disorders. Kluger et al. (1) proposed a new taxonomy for fatigue in neurologic diseases and suggested differentiating between fatigue as subjective sensation and fatigability as an objective change in performances. Here, we distinguish between cognitive and motor components, which can occur in isolation or in combination. Commonly, the subjective perception of fatigue is assessed using questionnaires (22), and the measurement properties of fatigue questionnaires in MS have previously been evaluated (23). The most frequently used instruments for measuring fatigue in MS patients are the Fatigue Severity Scale (FSS) (24), the Fatigue Assessment Instrument (FAI) (25), the Fatigue Impact Scale (FIS) (2), the Modified Fatigue Impact Scale (MFIS) (26), the Fatigue Scale for Motor and Cognitive Functions (FSMC) (27), and the Würzburg Fatigue Inventory in Multiple Sclerosis (WEIMuS) (28). Despite the reported prevalence of fatigue in MS and stroke, few studies used the same tools for assessing fatigue in these two conditions (compare also Lukoschek et al., in this special issue) (23). Moreover, in contrast to MS, there are no fatigue questionnaires that have been developed specifically for measuring fatigue after stroke (29). Often, the following instruments are used: the FSS (24), the Short-form 36/12 vitality questions (30), the Fatigue Assessment Scale (FAS) (31), and the Multidimensional Fatigue Symptom Inventory (MFSI) (14, 32). Overall, fatigue may be assessed quickly using fatigue questionnaires. However, these questionnaires are based on patients’ self-assessments and may be distorted (overestimation or underestimation) due to an inaccurate self-perception (33). Moreover, fatigue questionnaires capture patients’ general condition during a particular time period (33) and fatigue may also be quickly clinically assessed by physicians or physiotherapists. However, clinical experience suggests that an accurate identification of fatigue and non-fatigue depends on the experience of the therapists and physicians, and in some cases a clear diagnosis of fatigue is difficult. Especially, comorbidities (depression, sleep disorders, physical deconditioning, anemia, or side effects of medication) may cause similar symptoms (33). In these cases, the objective instrument can be extremely helpful for measuring fatigue. A correct diagnosis of fatigue is not only important to define optimal treatment but also when it is used as criterion for early retirement.

In the current study, we focused on the motor dimension of fatigability (here, we used the term motor fatigue as a synonym) in stroke and MS patients. The motor dimension of fatigability has previously been assessed in lower limbs using dynamometry in isometric contractions, sustained maximal contractions, repetitive maximal contractions, and walking as far as 500 m (34, 35) and in upper limbs using static and dynamic contraction tests (36–38) in MS patients. Hence, overall maximal force appeared to decrease either during repeated maximal contraction or during sustained contraction in MS patients. Furthermore, Severijns et al. (38) observed differences in sustained maximal hand grip contraction but not in dynamic contraction between healthy subjects and MS patients with high EDSS (≥6) (38). Schwid et al. (35) proposed that motor fatigue can be measured as a decline in strength during sustained muscle contractions (35). Similarly, Greim et al. (36) proposed that decreases in strength of maximal repetitive muscle contraction and/or decrease of walking speed can be used to measure motor fatigue objectively (36). Poststroke motor fatigue has previously been assessed in a few studies in upper and lower limbs using transcranial magnetic stimulation, dynamometry, and/or electromyography during the maximal voluntary contraction (MVC), sustained isometric contraction, submaximal contraction, and repetitive eccentric–concentric contraction (39–41). Knorr et al. (40) showed that during fatigue the silent period duration increased significantly in both upper limbs, whereas the motor evoked potential amplitude significantly increased only in the non-paretic limb (40). After fatigue, the reductions in the M wave, twitch peak torque, and MVC peak torque were observed in both limbs. Furthermore, the reduction in voluntary activation was greater in the paretic than in the non-paretic limb (40). Another study concluded that a reduction in work in high-intensity dynamic muscle activity may not be associated with a reduction in mean power frequency (39). Hu et al. (41) suggested that for identifying fatigue associated with neuromuscular transmission failure, the motor unit firing parameters firing rate, minimum inter-pulse interval, and maximum oscillation were more sensitive than the mean power frequency (41).

We recently developed the Fatigue index Kliniken Schmieder (FKS) as an objective tool for assessing motor fatigue in MS based on gait changes in a walking test on the treadmill (33). In this study, the subjects walked on a treadmill under different conditions: in a normal rested state and in an exhausted state or after 60-min walking. We measured the changes in acceleration patterns and acceleration variability of the feet during the walking test at the beginning and at the end of the walking test in MS patients and healthy subjects. Furthermore, in this study, we developed the FKS that is composed of these two components and which makes the distinction between fatigue and non-fatigue. The FKS described the changes in acceleration patterns and acceleration variability during the walking test on the individual level. The advantage of a walking test is that the entire musculature, especially the major muscle groups are required. This task is daily task-oriented and represents a complex movement with many degrees of freedom. In contrast to fatigue questionnaires, this test captures the current state of motor fatigue.

To date, it is still unclear if fatigue is specific to MS or at least to inflammatory disease or if it is an unspecific reaction of the brain after any kind of brain injury (1). The inflammatory etiology is supported by the fact that other inflammatory diseases such as sarcoidosis or cerebral vasculitis can be accompanied by serious fatigue. In stroke, fatigue may be related to reorganization or inefficient/suboptimal fiber tract connections or compensatory effort. Although we were not able to investigate different pathophysiological mechanisms directly by surrogate markers such as cytokines or tumor necrosis factor alpha or by different cerebral activation patterns, the intention of our study was to compare motor fatigue in patients with stroke and MS. This should facilitate better understanding limitations and needs of patients and more accurately define their goals for instance in rehabilitation. Therefore, the aim of our study was to investigate if the amount of change of the gait pattern during an exhausting physical task differs between stroke and MS patients. After propagating the test for identifying motor fatigue in MS (33), this investigation also should clarify if this test and the FKS are feasible for stroke patients and that a severe walking impairment in stroke patients does not necessarily cause a pathological FKS. Data of patients after stroke were collected and compared with previously published data (33) on 40 patients with MS and 20 healthy subjects.

## Materials and Methods

### Subjects

Ten patients who were admitted to a neurological rehabilitation clinic after stroke, met the inclusion criteria, and volunteered to participate between March and October 2012 were included in this study. Inclusion criteria were central hemiparesis affecting the leg, reduced walking capacity, and the ability to walk on a treadmill without aids or assistance. All stroke patients were chronic (time since the onset of stroke > 12 months). Hemiparesis was left sided in four patients and right sided in six patients. Eight patients had a proportional hemiparesis affecting arm and leg, and two patients were more affected in their legs. Three patients had a haemorrhagic infarction and seven patients an ischemic infarction. One infarct was located in the brainstem, one in the anterior cerebral artery (ACA), and eight in the middle cerebral artery (MCA). Two MCA infarcts showed additional involvement of the ACA.

Data from our previous study (33) involving 20 healthy subjects and 40 patients with definite MS according to the McDonald criteria (42) were used in this study. MS patients and control subjects were recruited between October 2011 and July 2012. The MS patients were admitted to a neurological rehabilitation clinic. Inclusion criterion for MS patients was the ability to walk on a treadmill without aids or assistance. There were no limitations regarding the disease course and disability levels. Subjects were excluded from the study if they had relapses within the preceding three months or received Fampyra^®^ (Fampridin; Biogen Idec Inc., 225 Binney Street, Cambridge, MA 02142). Healthy subjects were recruited from the local population and from clinic staff. Healthy subjects were excluded if they had any neurological or orthopedic disorders. In the previous study, the MS patients were classified into two groups based on the FKS: patients with a FKS > 4 were categorized as having motor fatigue (MS-F), and patients with a FKS ≤ 4 were categorized as having no motor fatigue (MS-NF). According to these criteria, 29 MS patients were in the fatigue group and 11 MS patients in the non-fatigue group.

All participants provided informed written consent prior to participation. The study protocol was approved by the University Ethics Committee and conducted in accordance with the Declaration of Helsinki.

### Questionnaires

At admission to the study, all subjects answered the Beck Depression Inventory II (BDI-II) to assess the level of subclinical depression (43). Self-reported physical function was assessed by patients using the physical functioning 10 subscale of the Short-form 36 (PF-10; SF-36) and four vitality questions of the SF-36 (44, 45). Vitality questions from the SF-36 have previously been suggested as measures of fatigue (46). These two assessments allowed for comparison of physical impairments and complaints about fatigue between groups.

### Experimental procedure

An exercise task and a functional test were carried out on two different days for each stroke patient. The exercise task included a walking test on a treadmill: patients walked either until they felt physically exhausted [17 – very hard, on the Borg scale (47)]; or for up to 60 min at 10% above their preferred speed or a maximum speed of 5 km/h on a level treadmill. The preferred walking speed was determined at an initial exam where each subject walked on the treadmill to familiarize them with the set-up. An important criterion was that the subjects were able to walk on a treadmill without aids or assistance. The walking speed was limited to a maximum of 5 km/h so that subjects stayed within a comfortable walking speed (48). The treadmill speed was kept constant throughout the test. The participants were repeatedly asked to rate their exhaustion on a Borg scale. The walking test was stopped 1 min after the patient reached 17 on the Borg scale or after 60-min walking on the treadmill. Kinematic gait data were measured for 1 min at the beginning of the walking test (t_1_) and for 1 min after reaching 17 on the Borg scale or for the final minute of 60 min (t_2_).

The functional test consisted of a 6-min walk test (6MWT) (49). The 6MWT is often used in clinical practice and has been frequently used for measuring the response to therapeutic interventions in various diseases. Heart rate was measured prior to and at the end of the walking test, and lactate concentration was measured prior to and immediately after walking. We used the 4 mmol/L lactate threshold originally described by Mader et al. (50).

### Technical equipment

The AS200 system (80 Hz; LUKOtronic, Lutz Mechatronic Technology e.U., Innsbruck, Austria) was used to record the gait data. This system consists of a three line-scanning camera system and 10 active markers attached bilaterally to the subjects’ body: centered on the margo medialis; the highest point of the ilium; the posterior aspect of the knee; on the shoes on top of the calcaneus and on the rod attached at the level of the ankle.

Videos were recorded with a HD digital camera synchronized with the motion analysis system (Exilim EX-F1, digital camera, Casio Computer Co. Ltd., Tokyo, Japan). Heart rate was captured using a chest strap and a gage (Garmin Forerunner 305, Garmin Ltd., KS, USA). Lactate levels in the blood were detected using a lactate analyzer and lactate strips (Arkray Lactate Pro LT-17810, Kyoto, Japan).

### Calculation of the Fatigue Index Kliniken Schmieder

**Figure 1:**
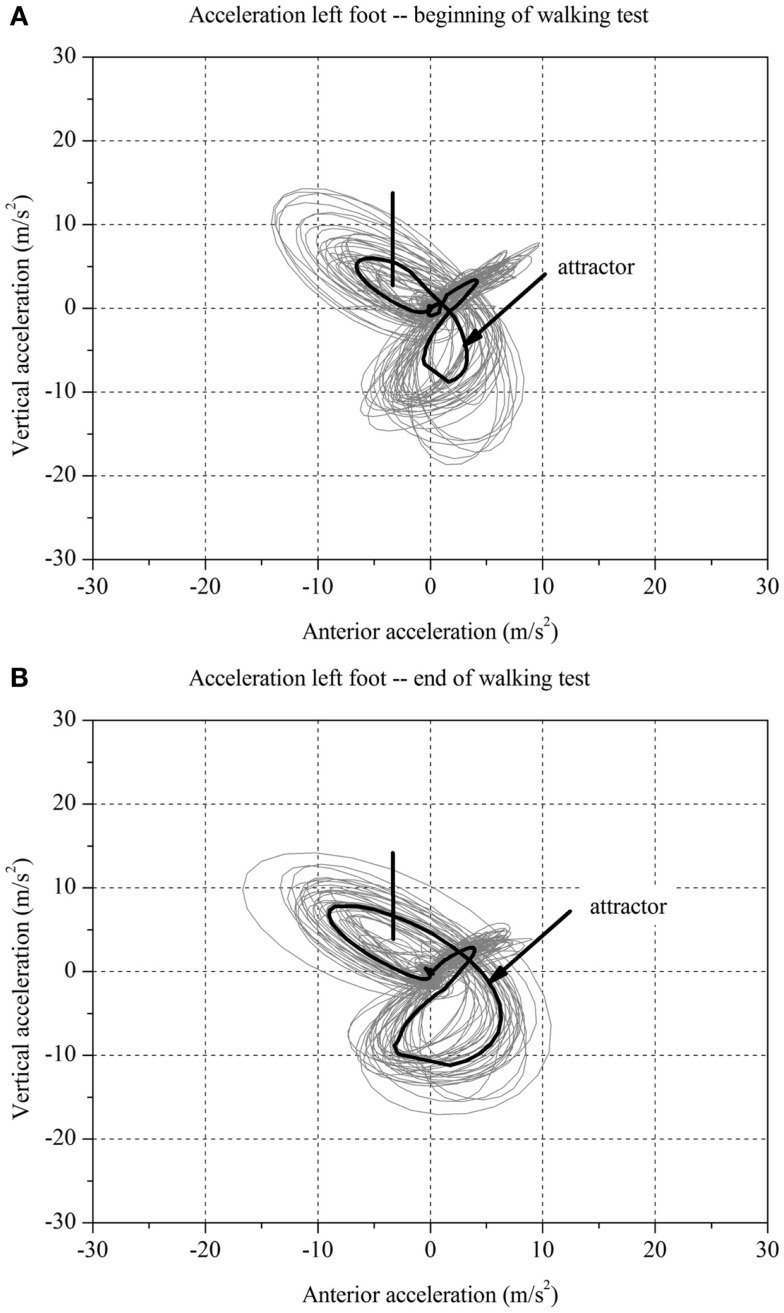
**Two-dimensional graph of the acceleration data of a subject’s left foot for one minute (A) at the beginning and (B) at the end of the walking test for one stroke patient with fatigue**.

**Figure 2:**
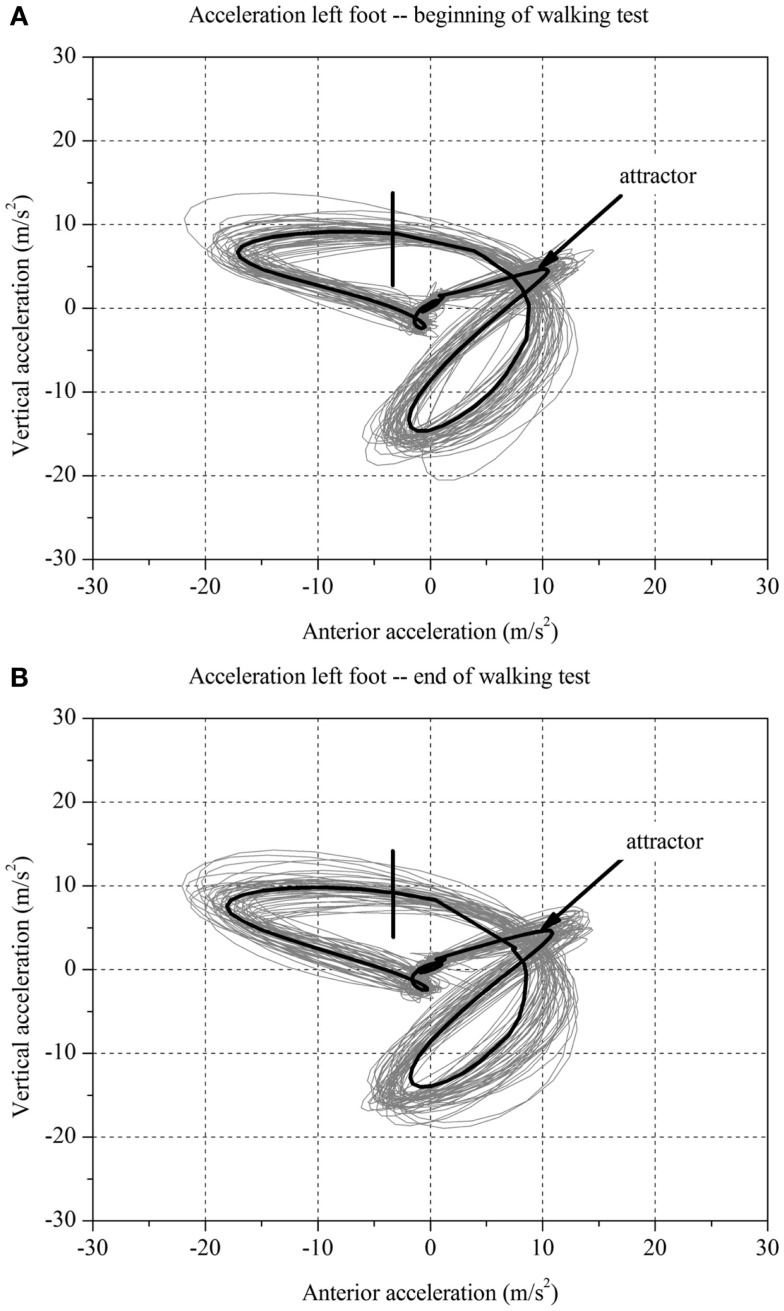
**Two-dimensional graph of the acceleration data of a subject’s left foot for one minute (A) at the beginning and (B) at the end of the walking test for one stroke patient without fatigue**.

For each stroke patient, the change in the movement pattern described by the attractor (δ*M*) and change in movement variability (δ*D)* of the acceleration of the feet between *t*_1_ and *t*_2_ were calculated (Figures 1 and 2A,B). This new method has recently been described in detail by Vieten et al. (51) and used to detect motor fatigue in patients with MS (33). The changes in movement acceleration patterns and variability were used as indicators of motor fatigue. It is well known that human walking in the absence of disturbances is characterized by a stable movement pattern and consistent movement control. We kept the walking situation unchanged throughout the walking test, and hence changes in attractor and movement variability indicated an alteration of the gait mechanism, which by ruling out other reasons, we identified as acute motor fatigue. The calculation of FKS was based on both feet. The FKS was defined as the changes in δ*M* and δ*D* between the beginning and the end of walking (51) and represented as

δF=δM⋅δD

The FKS was calculated for each stroke patient. These patients were then classified according to the FKS in a fatigue and non-fatigue group. This method allows analyzing fatigue on the individual patient level and on the group level. Based on FKS, stroke patients with FKS ≤ 4 were identified as having no motor fatigue (stroke-NF) and stroke patients with FKS > 4 were identified as having motor fatigue (stroke-F). The FKS cut-off of 4 was calculated in our previous study in the following order: first, using the group medians calculated using traditional methods (neurologist rating) to find the threshold between normal and fatigue (33). Second, the FKS of healthy individuals was used as a benchmark test. Third, all subjects were classified according to the FKS values into the fatigue and the non-fatigue groups.

### Conventional gait analysis

Spatial parameters were calculated: step length, step width, step height, maximum circumduction of the right and left leg, and medio-lateral sway of the upper body were calculated using three-dimensional co-ordinates of the active markers. This analysis allowed comparisons between different groups on the group level.

### Evaluation of the video recordings

The subjects’ movement patterns were recorded on videos captured during *t*_1_ and *t*_2_ from the side and from the back. Videos were evaluated by two experienced physiotherapists from the rehabilitation clinic. The order of the videos was randomized, and thus the physiotherapists did not know which video had been captured at the beginning and which at the end of walking test when attempting to correctly assign the videos to the corresponding time period. The physiotherapists did not evaluate the details regarding the modality of movement.

### Statistical analysis

Data of stroke patients were compared to those of MS patients and healthy control subjects (33). All statistical tests were performed using StatFree Version 8.0.0.9 (VietenDynamics, University of Konstanz, Germany) and Stata Version 11.0 (StatCorp LP, College Station, TX, USA). Differences in non-normally distributed parameters between groups were detected using Kruskal–Wallis test with Mann–Whitney *U* test as *post hoc* tests. For categorical variables, we used the χ^2^-test. Pearson correlation coefficients were used to detect significant associations between the changes in the movement pattern and changes in movement variability as well as between FKS and the results of BDI-II. The significance level for all statistical tests was set *a priori* to.05.

## Results

### Differences between stroke patients, MS patients, and healthy subjects

Table 1 presents descriptive characteristics for stroke patients, MS, and healthy subjects. Significant differences between stroke and MS patients were found for sex, age, height, and mass. Furthermore, the PF-10 and vitality score of the SF-36 differed significantly between the stroke and MS groups with a higher physical impairment and higher vitality level in stroke patients (*p* < 0.04 and *p* < 0.02, respectively). In contrast, no significant differences were detected between stroke patients and healthy subjects with the exception of age.

**Table 1:** **Mean (1 standard deviation) characteristics of participants**.

Characteristic	Stroke	MS	Healthy subjects	*p*-value
Sex male/female	7/3	13/27	9/11	0.03^a^
Age	51.6 (8.3)	45.9 (7.0)	43.1 (8.6)	0.03^a^
				0.01^b^
Height (cm)	177.2 (7.7)	171.4 (10.7)	173.4 (8.4)	0.04^a^
Mass (kg)	84.5 (16.5)	74.1 (15.6)	80.4 (21.3)	0.04^a^
SF-36, PF-10	16.3 (4.8)	21.0 (4.3)	Not collected	0.04^a^
SF-36, vitality	15.8 (2.2)	11.1 (3.5)	Not collected	0.02^a^
BDI-II (% of patients with depression)	20.0	65	15.0	0.02^a^
EDSS	Not applicable	3.4 (1.3)	Not applicable	
Disease duration (years)	8.3 (7.9)	10.8 (7.2)	Not applicable	

*MS, MS patients; SF-36, PF-10, 10 items of the physical functioning (ranging from 10 to 30, where low values indicate strong impairment, high values low impairment); SF-36, vitality scale, four items each ranging from 1 (low vitality/high fatigue) and to six (high vitality/low fatigue); BDI-II, Beck Depression Inventory II; EDSS, Extended Disability Status Scale ranging from 0 (no symptoms) to 10 (death through MS)*.

*^a^Significantly different between stroke and MS*.

*^b^Significantly different between stroke and healthy subjects. Only the significant differences are indicated*.

Based on the BDI-II questionnaire, one patient was affected by minimal depression and one patient was affected by slight depression in the stroke group. All other patients with stroke were not affected by depression. Moreover, 65% of MS patients and 15% of healthy subjects were affected by depression.

### Physical performance in stroke patients compared with MS patients and healthy subjects

Table 2 shows the physical performance in three groups. The stroke patients walked significantly slower than the healthy subjects (*p* < 0.001) and a shorter distance than MS patients and healthy persons (*p* < 0.01) in the walking test on the treadmill. Walking distance ranged from 1.0 to 3.4 km and walking speed ranged from 1.0 to 3.3 km/h in stroke patients. In MS patients, walking distance ranged from 0.2 to 5.6 km and walking speed ranged from 0.9 to 5.0 km/h. In healthy subjects, walking distance ranged from 5.0 to 6.0 km and walking speed was 5 km/h. The stated speed refers to the speed with which subjects walked on the treadmill after the familiarization phase and in which all data were collected. Some subjects walked slower in the familiarization phase and then they increased their speed. The important criterion was that the subjects do not walk over 60 min in the test. In the 6MWT, stroke patients walked a significantly shorter distance than the other groups (*p* < 0.001).

**Table 2:** **Mean (1 standard deviation) gait and physiological parameters of the walking test**.

Parameters	Stroke	MS	Healthy subjects	*p*-value Kruskal–Wallis test	*p*-value *Post hoc* test
Walking distance (km)	1.9 (0.9)	2.5 (1.6)	5.3 (0.3)	0.001	0.001^a^
Walking speed (km/h)	2.2 (0.8)	3.4 (1.4)	5.0 (0.0)	0.001	0.01^b^
					0.001^a^
6MWT (km)	0.30 (0.11)	0.51 (0.10)	0.68 (0.10)	0.001	0.001^b^
					0.001^a^
Lactate (mmol/L)
*t*_1_	0.7 (0.6)	1.1 (0.6)	0.8 (0.6)	0.04	0.02^b^
*t*_2_	0.2 (0.4)	0.6 (0.5)	0.6 (0.6)		
Heart rate (bpm)
*t*_1_	70.0 (10.8)	79.2 (11.0)	79.4 (20.7)		
*t*_2_	99.9 (13.2)	104.8 (16.8)	108.8 (20.8)		
Borg scale	14.0 (1.7)	16.0 (2.6)	10.0 (2.5)	0.001	0.001^b^
					0.001^a^

*Stroke, stroke patients; MS, MS patients; 6MWT, 6-min walk test*.

*^a^Significantly different between stroke and MS*.

*^b^Significantly different between stroke and healthy subjects*.

All subjects remained below the aerobic-anaerobic threshold (lactate concentration below 4 mmol/L) during the walking test and had a heart rate below the maximal heart rate. At the end of the test, the level of exertion on the Borg scale was significantly lower in stroke patients than in MS patients (*p* < 0.001). In contrast, stroke patients had greater levels of exertion than healthy subjects (*p* < 0.001).

### Conventional gait analysis in stroke patients compared with MS patients and healthy subjects

Significant group differences in gait parameters were observed at *t*_1_ and at *t*_2_ (*p* < 0.001). The results of the *post hoc* tests revealed that stroke patients had shorter step lengths and greater step widths than the other groups both at *t*_1_ and *t*_2_ (*p* < 0.001). Furthermore, the stroke patients had lower step height than the MS patients and healthy persons at *t*_1_ and *t*_2_ (*p* < 0.001). Circumduction with the right and left legs as well as the sway were significantly greater in the stroke group than in the other groups at *t*_1_ and *t*_2_ (*p* < 0.009).

### Video analysis

One physiotherapist correctly classified 6 of 20 (30%) and the other physiotherapist 8 of 20 (40%) videos of stroke patients indicating that they were correct just by chance and did not recognize increasing gait abnormality at the end compared to the beginning of the walking test. In contrast, the physiotherapists classified most of the videos correctly in the MS group 68 of 80 (85%) and 64 of 80 (80%), respectively. In healthy subjects, the physiotherapists properly classified 26 of 40 (65%) and 34 of 40 (85%) videos, respectively.

### Fatigue Index Kliniken Schmieder comparison between groups

**Figure 3:**
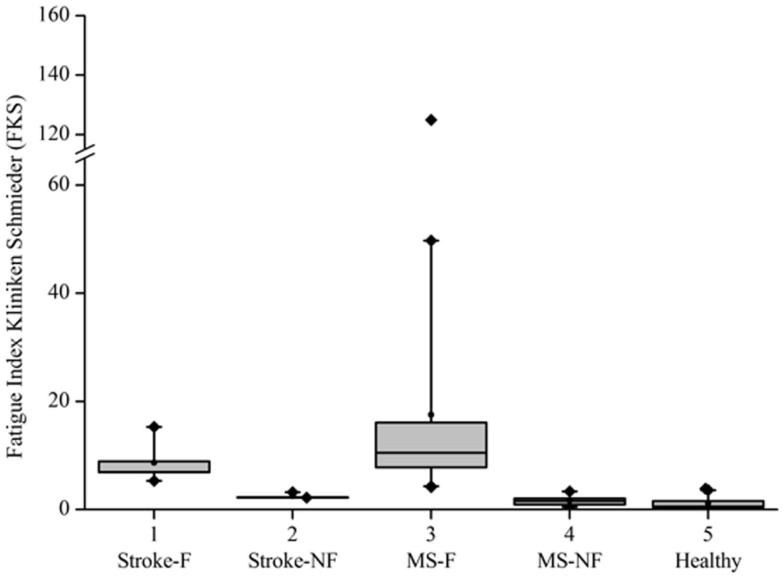
**Boxplot for FKS values in all groups**.

**Figure 4:**
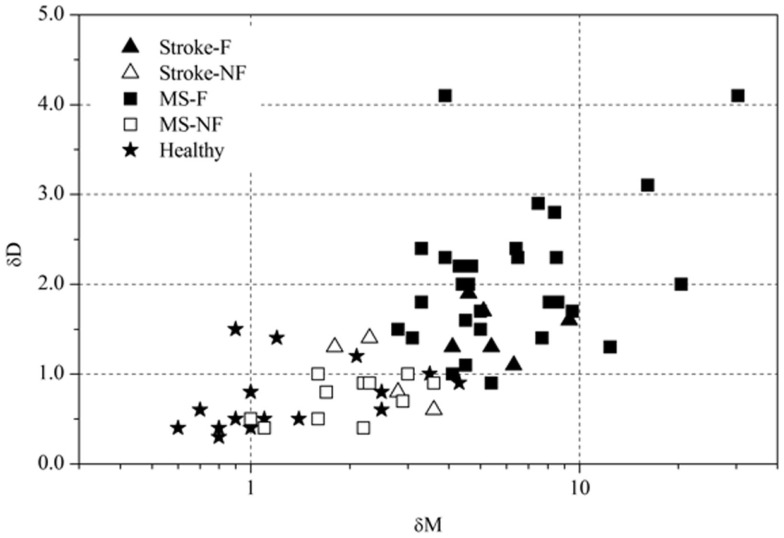
**Scatterplot between changes in movement pattern and movement variability**.

Based on the FKS scores, six stroke patients were classified into the fatigue group (stroke-F) and four patients into the non-fatigue group (stroke-NF). The FKS in the stroke-F group ranged from 5.3 to 15.3 (δ*M:* 4.1–9.3; δ*D:* 1.1–1.9) and in the stroke-NF group from 2.2 to 3.2 (δ*M:* 1.8–3.6; δ*D:* 0.6–1.4). The FKS in the MS-F group ranged from 4.2 to 125 (δ*M:* 2.8–30.4; δ*D:* 0.9–4.1) and in the MS-NF group from 0.5 to 3.4 (δ*M:* 1.0–3.6; δ*D:* 0.4–1.0). The FKS in the healthy subjects ranged from 0.3 to 3.9 (δ*M:* 0.6–4.3; δ*D:* 0.3–1.5) (Figure 3). The FKS differed significantly between stroke patients and healthy persons (*p* < 0.001) but not between stroke and MS patients (*p* = 0.44). In the subgroups, the FKS differed significantly between the stroke-F and the stroke-NF, MS-NF, and healthy groups (*p* < 0.01). Mean FKS in the stroke-F group was smaller than that in the MS-F group, but this difference did not reach statistical significance (8.7 versus 17.5; *p* = 0.18). In all groups, subjects with greater changes in movement patterns also showed greater changes in movement variability (*r* = 0.66, *p* < 0.001) (Figure 4). The differences in changes in movement patterns and changes in movement variability between groups corresponded to the differences in FKS between groups. Furthermore, FKS did not correlate significantly with the results of BDI-II (*r* = 0.27, *p* < 0.09).

## Discussion

The purpose of this study was to compare motor fatigue in stroke and MS patients by analyzing changes in movement patterns and their variability. In this pilot study, we observed no significant difference in FKS values between stroke and MS patients as well as in their subgroups: between stroke patients with fatigue symptom and MS patients with fatigue symptom. Hence, fatigue induced similar changes in the movement patterns and variability in both patient groups. Furthermore, the results of our study showed that the FKS can also be used in stroke patients for objectively measuring motor fatigue.

We intended to verify that severe walking impairment in stroke patients does not cause a pathological FKS. During the walking test on the treadmill, stroke patients rated their fatigue on the Borg scale significantly lower than the MS patients. Interestingly, despite lower perception of fatigue on the Borg scale, the stroke patients had greater physical impairment. All stroke patients had a hemiparesis affecting the leg. A higher level of impairment was observed using kinematic gait analysis, PF-10 of SF-36, and physical performance. Using conventional kinematic gait analysis of a few single stride cycles, we observed very clear differences in all gait parameters between the stroke patients and the other groups at *t*_1_ and *t*_2_. Generally, the stroke patients showed smaller step length, step height and greater step width, circumduction with the right and left leg, and greater sway compared to MS patients and healthy subjects. These results are in agreement with other studies (52, 53). The reduced step length and greater step width in stroke patients indicate an unsteady gait and the attempt to improve their stability to avoid falling while walking. The altered gait pattern already present at the beginning of the walking test on the treadmill, compared to the other groups, is presumably caused by the hemiparesis in this patient group. The reduction of walking speed and walking distance in stroke patients compared to the other groups as measured in our study are well established (53, 54).

Although stroke patients had higher physical impairment on PF-10 of SF-36 than MS patients, they showed greater vitality scores on the SF-36 than MS patients. These results point toward a conceptual and pathophysiological difference between impairment and fatigue. While it can be disputed whether or not fatigue should be rated as impairment, the neurological exam or the PF-10 of SF-36 do not assess fatigue.

The origin of peripheral or muscle fatigue is outside the central nervous system (CNS). For example, the peripheral fatigue can be caused by an increased blood lactate accumulation and hydrogen ions, accumulation of ammonia, loss of water, an accumulation of Pi (inorganic phosphate), and an accumulation of H+ ions in the sarcoplasm (55). There are several objective methods for measuring peripheral fatigue. Among others, muscle fatigue can be detected using surface electromyography (sEMG) and mechanomyography (MMG) (56). Previous studies investigated manifestations of fatigue in prolonged activities involving repetitive low force work tasks. In contrast to our study, they used task duration of more than 1 h with an intensity of 20% maximum voluntary contraction in an isolated movement with few active muscles (57). For example, they measured fatigue using electromyography of a descending part of the trapezius muscle. In our study, walking is a complex movement with involvement of many muscle groups and several degrees of freedom. Based on the results of our previous studies, we expected that patients with fatigue would be exhausted in less than 60 min (33, 58). One of the most popular cost-efficient and quick measurement of muscle fatigue is the analysis of blood lactate during exhaustive exercises. We used this method in our study. All subjects walked on the treadmill without reaching their lactate threshold, which reflects the rate at which a person can work aerobically without accumulation of acid substances associated with muscular fatigue (59). However, some patients have reached exhaustion as these patients reported 17 (very hard) on the Borg scale and/or the FKS was >4. None of the healthy persons reached exhaustion in the walking test determined using the Borg scale and the FKS. Hence, it seems unlikely that motor fatigue was not associated with muscular fatigue.

A strong relationship between depression and fatigue has been described in both patient groups (3, 18). Moreover, depression is considered one of the most confounding factors associated with fatigue; it can be hard to disentangle depression and fatigue in a patient. In the present study, the depression was more common in MS groups than in the stroke or healthy subjects. Epidemiological studies reported that depression is common in MS with annual prevalence rates as high as 20% and a lifetime prevalence of up to 50% (60–62), which is approximately three times higher than in healthy people (61). Approximately one-third of all patients with stroke experience depression symptoms and the prevalence only slightly decreases within the first 2 years after stroke (63, 64). In our study, the FKS did not correlate with BDI-II. The FKS is an important tool for detecting motor fatigue objectively and independent of the presence or absence of depression.

It may be speculative and beyond the scope of the present investigation, but the motor fatigue in stroke and MS patients probably suggests different underlying pathophysiological mechanisms. Ischemic lesions occur according to the all-or-nothing principle: if oligemia causes an ischemic lesion, it results in a complete lesion of the tissue finally ending up in the chronic stage as a substantial cyst (simply speaking as a hole in the brain). Fatigue in this case may be related to compensation or use of alternative, less efficient, or reorganized pathways. Inflammation in MS might cause demyelination or partial impairment of neural pathways. Neuronal function may be partially preserved, but under high demand or long or highly repetitive requirements function might slowly decline. Further or additional compensation does not seem to be possible, and it is unclear if this is due to loss of K^+^ as suggested in the literature explaining the function of 4-aminopyridine (65). Completely different pathomechanisms may be related to inflammatory substances such as cytokines or tumor necrosis factor alpha (TNF-α) (Hacken et al., this special issue) (8). Increased cytokines, however, are not a prominent finding in the liquor of chronic stroke patients, and hence fatigue is expected to have a different pathomechnism in stroke. Different pathomechanisms of fatigue would require different treatment options (8, 66). For instance, compensation in stroke patients may be enhanced by training, and electric failure in MS lesions may be ameliorated by substances such as 4-aminopyridine or inhibitors of TNF-α (67, 68).

Most standardized fatigue questionnaires are based on patients’ self-assessments and often used for rating fatigue symptoms. However, because these questionnaires are based on the patients’ subjective impressions, they may be distorted because of an inaccurate self-perception. Currently, most of the fatigue questionnaires are disease specific and have been specifically developed to assess fatigue in MS (29). Elbers et al. (23) recommended the FSMC for the multidimensional assessment of fatigue in MS patients (23). In contrast, the FSS is the most commonly used instrument to measure fatigue in stroke patients (69), which was also recommended by Elbers et al. (23). Since most of the motor scores are disease specific, it is not easy to compare the degree of impairment in stroke and MS patients. For instance, the Motricity Index (70), the Fugl-Meyer test (71), or Rivermead Motor Assessment (72) are evaluated for stroke, whereas the application of the Expanded Disability Status Scale (73) is restricted to MS, and there is no common measure for both entities. To overcome this difficulty, we used the Physical Functioning Scale of the SF-36 to assess daily life motor activities and their restrictions. This allowed for some rough comparison of motor impairment and disabilities in daily life. Currently, there is a validated scale for fatigue in both MS and stroke patients (74), which was not available at the time of data collection.

The estimation error may occur in the clinical assessment of the patient by physicians and physiotherapists. Some patients are hard to classify into fatigue and non-fatigue groups based on patient’s survey and traditional clinical tests carried out by physicians and therapists. The results of the FKS largely agreed with the results of the video analysis in MS patients. The physiotherapists assigned videos of the beginning and end correctly in 80–85% of MS patients. Such classification was difficult for stroke patients and healthy subjects. In general, the MS patients have almost an unremarkable gait pattern at the beginning of walking. In the state of fatigue, the gait changed greatly. Thus, it can be clearly seen in most cases. However, it depends on the experience of the physiotherapist. In contrast to MS, the stroke patients had an impaired gait pattern at the beginning of walking test. All stroke patients had a hemiparesis and hence an abnormal gait pattern at both time points. It is possible that the raters cannot be distinguishing between the abnormal gait characteristics caused by the hemiparesis and those caused by motor fatigue. This could lead to difficulties to assess the changes in gait pattern. Even if this evaluation was very successful for these cases, the analysis is subjective and depends on many factors and particularly on the therapists’ experience. These results emphasize the importance of an objective measure of motor fatigue that is independent of the subjective assessment of a rater.

The FKS is an objective measure. As acknowledged above in many cases, a neurologist can detect the presence of fatigue in patients with MS using “classic” instruments. However, in some cases, a physician cannot be sure of the diagnosis of the fatigue syndrome, and in these cases, the FKS can be extremely helpful for objectively measuring motor fatigue. The correct diagnosis of fatigue is especially important when it is used as criterion for early retirement emphasizing the relevance of this test. For example, the most important differential diagnosis may be depression. In some instances, it may not be easy to disentangle both phenomena. Treatment may be similar involving antidepressive agents, increasing regular physical activity, acceptance of limitations, energy conservation programs, etc. However, the patient will feel more accepted and understood, if the therapist and neurologist are able to discriminate, explain, and treat different components of his complex symptom. Moreover, the FKS can be used both for diagnosis and for the evaluation of the course of treatment.

## Conclusion

Using FKS, a new and objective tool for identifying and quantifying motor fatigue, we found that fatigue was similarly pronounced in both patient groups. We observed that a more severe walking impairment in stroke patients at baseline is not associated with a pathologically higher FKS. The objective assessment of motor fatigue via the FKS allows the comparison of motor fatigue between stroke patients, MS patients, and healthy persons.

## Conflict of Interest Statement

The authors declare that the research was conducted in the absence of any commercial or financial relationships that could be construed as a potential conflict of interest.
